# CLARIFYING THE ROLE OF RESILIENCE IN NONHUMAN PRIMATE AGING USING A BABOON CELL MODEL

**DOI:** 10.1093/geroni/igac059.1612

**Published:** 2022-12-20

**Authors:** Daniel Adekunbi, Cun Li, Peter Nathanielsz, Adam Salmon

**Affiliations:** University of Wyoming, Laramie, Wyoming, United States; University of Wyoming, Laramie, Wyoming, United States; University of Wyoming, Laramie, Wyoming, United States; The University of Texas Health Science Center at San Antonio, San Antonio, Texas, United States

## Abstract

Aging is associated with defects in homeostatic maintenance. Delineating the dynamics of the homeostatic network is challenging in vivo and often limited by endpoint measurements. However, we have developed carefully controlled translatable cellular models to bridge some of these challenges using primary cells obtained from male and female baboons across the life course (4 to 21 years). We recently published findings showing that donor age corresponds to loss of cellular resilience to oxidative stress in males but not in females. Responses to thapsigargin-induced endoplasmic reticulum (ER) stress were also age and sex dependent, though with different pattern than that of oxidative challenge. Young females alone were vulnerable to ER stress, while young males and old females were not affected. These data highlight that primary cells retain donor characteristics regarding molecular mechanisms associated with aging including those dependent on sex. To further investigate these mechanisms, we investigated whether the proteostatic machinery contributes to cellular resilience outcome following ER stress. In female donors, we find significant decrease in 20 S proteasome activity and expression of its catalytic subunit (PSMB 8) in female-derived fibroblasts under basal or ER stress conditions when compared to males. ER oxidoreductin 1 (ERO1) as well as autophagy marker, LC3-II/I ratio, were significantly higher in young females compared to young males during ER stress. These data highlight the propagation of sexual dimorphisms in cellular resilience at the molecular level and suggest that sex differences in maintenance of proteostasis contributes to the vulnerability of young females to ER stress.

